# Spacio-Linear Screening for Ligand-Docking Cavities in Protein Structures: SLAM Algorithm

**DOI:** 10.3390/life16020285

**Published:** 2026-02-07

**Authors:** Julia Panov, Alexander Elbert, Dean S. Rosenthal, Moshe Levi, Konstantin Chumakov, Raul Andino, Leonid Brodsky, Hanoch Kaphzan

**Affiliations:** 1 Tauber Bioinformatics Research Center, University of Haifa, Haifa 3103301, Israel; 2 Sagol Department of Neurobiology, University of Haifa, Haifa 3103301, Israel; 3 Department of Biochemistry and Molecular & Cellular Biology, School of Medicine, Georgetown University, Washington, DC 20057, USA; 4 Center for Biological and Biomedical Engineering, Georgetown University, Washington, DC 20057, USA; 5 Department of Microbiology, Immunology and Tropical Medicine, George Washington University, Washington, DC 20037, USA; 6 Department of Microbiology and Immunology, University of California San Francisco, San Francisco, CA 94158, USA; 7 Department of Evolutionary and Environmental Biology, University of Haifa, Haifa 3498838, Israel; 8 School of Medicine, University of Haifa, Haifa 3103301, Israel

**Keywords:** binding site similarity, structural alignment, 3D screening, protein ligand docking

## Abstract

Identifying structurally similar ligand-binding sites in unrelated proteins can facilitate drug repurposing, reveal off-target effects, and deepen our understanding of protein function. A number of tools were developed for structural screening, but many of them suffer from limited sensitivity and scalability. Using a data bank of crystallized protein structures, we aimed to discover novel protein targets for a ligand by leveraging a known ligand-binding query protein with a resolved structure. Here, we present SLAM (Spacio-Linear Alignment of Macromolecules), a novel alignment-based algorithm that detects local 3D similarities between ligand-binding cavities or protein-exposed surfaces of query and target proteins. SLAM encodes spatial substructure neighborhoods into short linear sequences of physicochemically annotated atoms, then applies pairwise sequence alignment combined with distance-correlation scoring to identify high-fidelity structural matches. Benchmarking using the Kahraman-36 dataset demonstrated that SLAM outperforms the state-of-the-art ProBiS algorithm in true-positive rate for predicting ligand-docking compatibility. Furthermore, SLAM identifies candidate ligands that may inhibit functionally critical domains of CRISPR-Cas proteins and predicts novel binding partners of toxic per- and polyfluoroalkyl Substance (PFAS) compounds (PFOA, PFOS) with plausible mechanistic links to toxicity. In conclusion, SLAM is a robust computationally efficient and flexible structural screening tool capable of detecting subtle physicochemical compatibilities between protein surfaces, promising to accelerate target discovery in pharmacology and elucidate protein–ligand interactions in environmental toxicology.

## 1. Introduction

Identifying similar ligand-binding cavities in structurally unrelated proteins has broad implications for modern drug discovery, including drug repurposing, predicting off-target effects, and elucidating the mechanisms of drug–target interactions [1]. Although protein cavities that bind the same class of small molecules often exhibit considerable structural diversity [2], successful ligand binding typically requires a certain degree of geometric and physicochemical complementarity. Consequently, structurally similar cavities are more likely to bind ligands with comparable physicochemical contact profiles [3].

Comparing structural similarities between ligand-binding cavities in different proteins enables the assessment of whether a known ligand (‘probe ligand’) of a ‘query protein’ can bind to a structurally similar cavity in another (‘target’) protein. Moreover, since a probe ligand may also interact with alternative surface patches on the target protein, comparing the ligand-binding pockets of the query protein to the solvent-exposed regions of the target protein can uncover novel binding sites. These complementary approaches for screening ligand-binding pocket similarity are instrumental for identifying potential drug targets and elucidating protein functions, ultimately accelerating drug discovery.

A range of computational methods have been proposed to identify similarities between the ligand-binding pockets in protein structures that are available in the Protein Data Bank (PDB). The PDB is a comprehensive repository of experimentally resolved biomolecular structures and currently contains over 200,000 entries. Notably, approximately 70% of these entries involve protein–ligand complexes [4], in which small molecules or drug-like compounds are bound to proteins. As a result, algorithms for analyzing structural similarities and evaluating ligand-docking potential frequently rely on the PDB as a foundational resource for identifying and characterizing potential drug targets.

A variety of algorithmic approaches are available for identifying local similarities between the binding potential of protein structures. These methods span from traditional sequence and structural alignment techniques to alignment-free strategies that evaluate pocket similarity based on integral physicochemical and geometric characteristics. For example, amongst the alignment-based algorithms are Lamarckian Genetic Algorithms (LGAs), which represent binding pockets through sorted interatomic distances, thus encoding their shape and chemical properties [5]. Similarly, fpocket [6] evaluates pocket similarity by analyzing the shapes of the molecular surfaces and their electrostatic potentials. More recently, 3D Zernike descriptors have been employed to quantify and compare pocket shapes [7]. Alongside these alignment-based algorithms, alignment-free approaches have been recently developed and are becoming popular, including the use of convolution neural network-based image recognition in Deeply Tough [8] and the topological water network analysis utilized by the TWN-RENCOD technique [9]. Furthermore, additional algorithms based on graph theory and clique detection such as ProBiS [10] and the PDBspheres method [11], the latter of which uses the exhaustive library of protein structure regions (‘spheres’) adjacent to complexed ligands derived from the PDB, have been utilized to compare similarities between binding sites.

However, despite the aforementioned advancements, existing methods still suffer from limited specificity and sensitivity, highlighting the need for complementary and alternative approaches. To address this challenge, we developed SLAM (Spacio-Linear Alignment of Macromolecules)—a novel algorithm designed to evaluate the compatibility of a target protein cavity with a given probe ligand.

**Figure 1:**
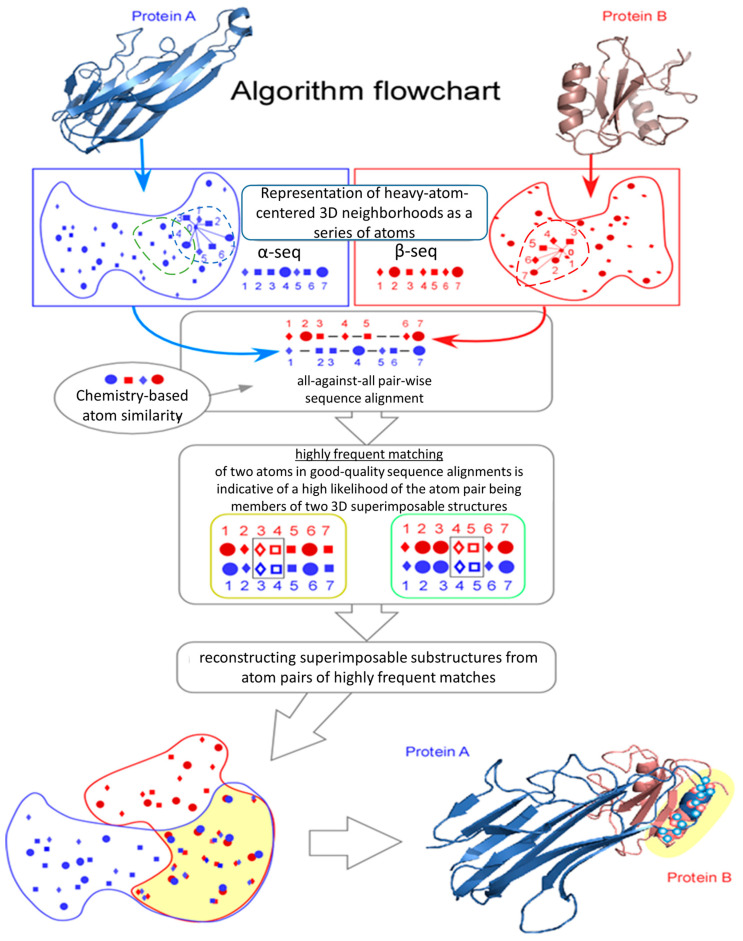
Flowchart of the SLAM (Spatio-Linear Alignment of Macromolecules) algorithm for identifying local 3D similarities between protein structures. SLAM detects local structural similarities by representing neighborhoods of atoms in protein–ligand binding cavities as short, linearly ordered atom sequences and comparing them using sequence alignment methods. The algorithm proceeds through the following computational steps: (1). Selection of candidate neighborhood centers: Each heavy atom (non-hydrogen) in proteins A and B is treated as a potential center (α or β) of a local neighborhood. (2) Generation of local atom sequences: For each center, a neighborhood is constructed by selecting 7–11 nearby atoms, sorted by increasing distance from the central atom, to form linear sequences (α-seq and β-seq). (3) Annotation with physicochemical properties: Each atom in each of the sequences is annotated with descriptors capturing its chemical environment and the properties of its parent amino acid (see Section 2.2.1 for details). (4) Pairwise sequence alignment: The Needleman–Wunsch algorithm is used to align α-seq and β-seq based on physicochemical similarity and gap penalties. Since the sequences are defined by 3D proximity, this process implicitly aligns the local 3D regions (‘a’ in protein A to ‘b’ in protein B). (5) Frequency filtering of atom matches: Atom pairs that frequently occur in high-scoring alignments are retained as candidate 3D matches (Section 2.2.2). (6) Expansion into larger 3D substructures: Hierarchical clustering is used to merge consistent atom pairs into larger aligned substructures, optimizing the Pearson correlation of interatomic distances (see Section 2.2.3). (7) Final scoring of 3D alignments: The final SLAM score (Ncorr5) integrates alignment size (N), the correlation of interatom distances, and the overall physicochemical similarity between matched atoms (Section 2.2.4).

In brief, SLAM begins with a PDB structure of a probe ligand–protein complex and extracts the ligand-binding cavity (pocket or ligand neighborhood). Importantly, SLAM requires as input at least one resolved protein–ligand complex and does not perform de novo identification of ligands or binding sites in the absence of such a reference structure. It then performs 3D alignment against either the pre-extracted ligand-binding cavities or extracted solvent-exposed surface patches of the target proteins (Figure 1). The 3D structures of both the query and target regions are parsed into overlapping local neighborhoods of central atoms that are typically 7–11 atoms in size. Each seven-atom neighborhood is encoded as a linear sequence of its seven heavy (non-hydrogen) atoms ordered according to their distance from the neighborhood’s central heavy atom and annotated with each atom’s physicochemical properties. These sequences are compared using pair sequence alignment based on the Needleman–Wunsch algorithm [12], with atom substitution scores reflecting the physicochemical similarity of atoms. Cumulative input from higher-scoring alignments reveals matching atom pairs between the probe and target regions. These matched atom pairs are then expanded into larger structural motifs by optimizing the correlation of interatomic distances and maximizing the number of aligned atoms. This representation enables fast and efficient comparison of local 3D substructures, enhancing the identification of potential ligand-binding patches. Following structural similarity assessment, the probe ligand is docked into the aligned region of the target protein using AutoDock Vina (autodock_vina-1.2.5) [13] and its binding affinity is evaluated through free-energy calculations.

To evaluate SLAM, we compared its performance to that of the widely used ProBiS algorithm [10] and showed that SLAM identifies a greater number of potential binding partners for these ligands with a high likelihood of binding to the probe ligand, as supported by AutoDock Vina free-energy calculations.

In addition, we demonstrated SLAM’s applicability across diverse biological contexts in a two-way manner, first for predicting ligands of CRISPR-Cas proteins and second for finding protein binding partners of per- and polyfluoroalkyl Substances (PFASs).

Overall, our SLAM approach provides a flexible and efficient method for screening structural protein databases across diverse scenarios for identifying local structural similarities and discovering novel binding partners for a probe ligand. It can be applied to experimentally resolved structures in the PDB, as demonstrated in this study, as well as to AlphaFold-predicted protein models. The SLAM software (v1) is freely available at https://github.com/TauberBioinformatics/SLAM.

## 2. Materials and Methods

### 2.1. Overview of SLAM Algorithm

This section introduces the conceptual foundation of the SLAM algorithm, which detects local 3D similarities between proteins by aligning physicochemically annotated atomic neighborhoods in a linear manner. These linear alignments reflect the 3D geometrical and physicochemical similarity of the compared neighborhoods.

**Figure 2:**
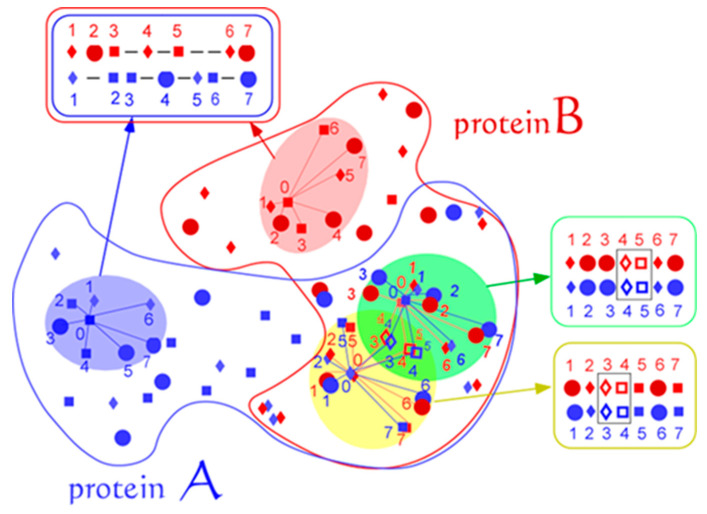
Atom pair detection frequency as an indicator of substructure superimposability. Schematic visualization of two protein structures, A (blue) and B (red), with atoms of different chemistries represented by distinct shapes. Structurally similar regions between the two proteins are superimposed. The figure illustrates four atomic neighborhoods centered on labeled atoms (‘0’): The blue- and red-framed neighborhoods originate from dissimilar regions in proteins A and B, respectively, and exhibit poor linear alignment due to structural divergence. The yellow- and green-framed neighborhoods are from structurally similar regions and yield high-quality linear alignments. Within the conserved neighborhoods, atom matches such as atom 3 and 4 (in the yellow region of protein A) and atoms 4 and 5 (in the green region of protein B) recur across multiple high-scoring alignments. These frequently detected atom pairs are used by SLAM to infer structurally superimposable substructures. Thus, the frequency of atom pair detection across good-quality alignments serves as a robust indicator of local 3D similarity between protein regions.

Let us assume that proteins A and B partially overlap with each other in 3D space, so that their superimposed atoms are physicochemically similar to each other (Figure 2). And let us arbitrarily select a pair of superimposed atoms (blue and red diamond shapes denoted as ‘0-0’ in Figure 2, yellow area) to be the centers of two superimposed neighborhoods containing an additional seven atom matches (1-1, 2-2, …, 7-7) from the two proteins (Figure 2, yellow area). Since the neighborhoods are superimposable, the set of distances between each of the atoms one-through-seven and the corresponding reference atom ‘0’ in the two proteins are similar.

If the seven-atom sets from each protein are sorted by increasing distance from a reference atom (‘0’), the linear alignment of these atomic sequences from two superimposable neighborhoods will exhibit strong chemical correspondence (Figure 2, alignments in yellow and green frames). In contrast, aligning sequences from non-superimposable neighborhoods will result in numerous mismatches or gaps (Figure 2, blue–red frame). Alternatively, identifying a good pairwise alignment of atom sequences serves as an indicator of a good match between the corresponding neighborhoods in three-dimensional space.

In reality, the centers of the overlapping neighborhoods (‘0-0’) between the two proteins are not known a priori. Therefore, each of the heavy atoms (non-hydrogen) within the protein substructure (e.g., a ligand-binding cavity or solvent-exposed protein surface) is systematically treated as the center of a local neighborhood. For each such center, an eight-atom neighborhood is constructed by selecting the seven nearest neighboring atoms, resulting in a linear sequence of seven atoms ordered by their increasing distance from the central atom. In this way, the neighborhoods partially overlap. The algorithm then performs all-against-all pairwise linear alignments between the atom sequences of neighborhoods derived from proteins A and B. Atom pairs located within structurally superimposable regions of proteins A and B are likely to recur across multiple sequence alignments (Figure 2, marked atom pairs in the yellow and green areas and frames). Thus, structurally superimposable regions of the two proteins are identified by detecting atom pairs that occur frequently across multiple pairwise linear alignments. Once atom pairs from proteins A and B are identified based on their frequent appearance in high-quality sequence alignments, the next step is to expand these matches into larger superimposable regions. To do this, previously identified atom pairs are grouped using a hierarchical clustering approach, where clusters are merged only if the Pearson correlation coefficient (r) between interatomic distances within each protein exceeds a predefined threshold (r = 0.85). This ensures that the spatial arrangement of atoms in both proteins remains structurally consistent.

### 2.2. SLAM Algorithm: Step-by-Step Workflow

The SLAM algorithm follows a set of computational steps to identify local 3D similarities between two protein structures (Figure 1):

Step 1: Selection of Candidate Neighborhood Centers

Any two heavy atoms (non-hydrogen) in two protein structures A and B are considered as possible centers *α* and *β* of a compared local neighborhood from A and B.

Step 2: Generation of Local Atom Sequences

For each selected center, a neighborhood is formed by selecting a small number of atoms (typically 7–11) located in close proximity to the corresponding centers (*α* or *β*). Atoms of each neighborhood are arranged in a linear sequence sorted by increasing distance from the corresponding central atom. This creates two linear sequences of atoms: *α*-seq and *β*-seq.

Step 3: Annotation of atoms with Physicochemical Properties

Each atom in the sequences is annotated with multiple physicochemical features, reflecting its chemical environment and the properties of the amino acid to which it belongs (for details of physicochemical atom similarity measure see Section 2.2.1).

Step 4: Pairwise Sequence Alignment

Following atom annotation, the Needleman–Wunsch sequence alignment algorithm [12] is then applied to align these sequences, accounting for both physicochemical similarity and gap penalties. The 3D geometrical consistency of aligned atoms is ensured because both *α*-seq and *β*-seq are constructed and ordered based on the 3D atoms’ proximity to their respective central atoms. As a result, the linear sequence alignment serves as implicit, flexible 3D alignment of small local atomic neighborhoods (‘a’ in structure A and ‘b’ in structure B).

Step 5: Frequency Filtering of Atom Matches

Atom pairs that recur frequently in high-scoring linear alignments are selected as candidate matches for atom-to-atom 3D superimposition (for details of atom pairing see Section 2.2.2).

Step 6: Expansion into Larger 3D Substructures

Hierarchical clustering is used to merge atom-to-atom 3D superimpositions into larger local 3D alignments. This merging process optimizes the Pearson correlation of interatomic distances between atoms in substructures A and B of the larger local 3D alignment, with the correlation required to exceed a specified threshold (for details of reconstruction of superimposable substructures see Section 2.2.3).

Step 7: Final Scoring of 3D Alignments

The final score of the 3D alignment (‘Ncorr5’) is calculated based on three factors: (i) the alignment length (N), (ii) the Pearson correlation of the interatom distances between aligned substructures A and B, and (iii) the overall correspondence of the physicochemical properties of the atom pairs across all of the small linear alignments within the 3D alignment (for details of the scoring of 3D alignments see Section 2.2.4).

#### 2.2.1. Physicochemical Similarity Measure for Atom-to-Atom Comparison

Linear sequences of atoms (*α*-seq and *β*-seq) from the two proteins (A and B) are aligned by matching similar atoms to each other. Two criteria were used to assess atom similarity for this alignment.

The first criterion reflects the similarity between the amino acids to which the two atoms belong. We evaluated several approaches for defining amino acid similarity, including the PAM250 substitution matrix [14] which estimates the likelihood of one amino acid replacing another during evolution and thus serves as proxy for their similarity. Our comparisons indicated that the results only weakly depended on the specific choice of amino acid similarity matrix. Notably, a reduced alphabet of seven amino acid groups [15], rather than the full set of 20, was sufficient for capturing relevant amino acid similarities. Consequentially, we adopted the PAM250 matrix in conjunction with the reduced seven-amino acid alphabet as the equivalence measure.

The second similarity criterion was the chemical similarity between the atoms, defined using the DABE key [16], which encodes four properties: potential hydrogen bond donor, potential hydrogen bond acceptor, bulkiness, and Sybyl electropositivity [17]. Each atom was assigned a four-digit binary string representing the presence (1) or absence (0) of these properties. For example, an atom that can function as both a hydrogen bond donor and acceptor, is bulky, and its Sybyl-electropositive would be encoded as (1,1,1,1). Hydrogen bonding properties were assigned based on annotations from the Sybyl software (https://sybyl.com/) suite [18].

The ‘bulkiness’ feature of an atom, *i*, was determined based on its volume and the volumes of its nearest neighboring atoms, *j*, as estimated from their respective Van der Waals radii, *w_i_* and *w_j_* respectively. An atom was classified as bulky if

$${w_{i}}^{3}+\sum_{j}{w_{j}}^{3}>10{\dot{A}}^{3}$$

The nearest neighbors of an atom were defined based on Van der Waals contacts, considering only heavy atoms. Van der Waals radii were obtained from the Sybyl software suite [18].

Electronegativity, used here as a proxy for atomic hydrophobicity, was assigned based on Pauling’s scale [19] in combination with Sybyl atom types. An atom was classified as Sybyl-electropositive if both its own and its neighboring atoms’ Pauling electronegativities were ≤2.5. Notably, both bulkiness and Sybyl electropositivity are conformation-dependent properties, and changes in protein conformation may alter the classification of atoms with respect to these features.

Of the 16 possible combinations of the DABE key, only 10 are observed in proteins. Chemical similarity between two atoms was quantified by counting the number of shared properties, yielding a score ranging from 0 to 4. For example, the similarity between two atoms with DABE strings (1,1,0,0) and (1,1,1,0) is 3, as they share identical values in the first, second, and forth positions. The total alignment score for a matched atom sequence was calculated as the sum of the amino acid similarity weights and the DABE key similarity scores. To ensure compatibility, the amino acid similarity weights were scaled to match the range of the atom–atom similarity scores.

#### 2.2.2. Atom Pair Detection Frequency as an Indicator of Substructure Superimposability

Let atom a_0_ be the center of a neighborhood in protein A, and atom b_0_ be the center of a neighborhood in protein B. Suppose these neighborhoods are superimposable (Figure 2). Due to their structural similarity, the sorted sequence of atoms a_0,1_, …, a_0,n_ in protein A is expected to align well with the corresponding sequence b_0,1_, …, b_0,n_ in protein B (e.g., Figure 2, with green and yellow neighborhoods showing good atomic superimposition). In contrast, even if two atoms a_0_ and b_0_ are chemically identical to each other, but reside in non-superimposable neighborhoods, their associated atom sequences will typically differ substantially from each other and cannot be aligned effectively (Figure 2, blue and red neighborhoods of proteins A and B).

When the similarity between atom sequences a_0,1_, …, a_0,n_ and b_0,1_, …, b_0,n_ reflects a true superimposition of substructures within the proteins A and B, additional sequence similarities are likely to be observed in neighboring regions (e.g., the yellow-highlighted neighborhood in Figure 2). Furthermore, since many neighborhoods partially overlap, atom pairs from superimposable regions are likely to recur across multiple linear alignments. For instance, the atom pairs labeled 4 and 5 in the green-highlighted neighborhood also appear as pairs 3 and 4 in the yellow neighborhood (Figure 2).

We perform an all-against-all sequence alignment of the atom sequences corresponding to neighborhoods in proteins A and B. As discussed above, matching pairs from structurally similar regions are likely to recur across multiple high-quality sequence alignments. In contrast, atom pairs that do not correspond to structurally similar positions are expected to appear infrequently, if at all, and typically only in low-scoring alignments. It is highly unlikely for a pair of non-matching atoms to occur repeatedly by chance in many high-quality sequence alignments. Therefore, the frequency with which a given atom pair appears in high-scoring alignments serves as an indicator for its likelihood to be part of structurally similar neighborhoods.

This frequency-based filtering creates a separation between two classes of alignments: one consisting of random or background matches characterized by a distribution of low scores, and another consisting of high-scoring outliers that reflect meaningful structural similarity. A threshold is then applied to distinguish between these two distributions, enabling the identification of atom pairs that likely correspond to true structural matches.

In practice, we perform an all-against-all Needleman–Wunch alignment of an atom sequence set from protein A against that of protein B to identify similar local neighborhoods. A superimposition likelihood score is then assigned to each atom pair (a,b) based on the cumulative quality of the linear pairwise alignments in which the pair (a,b) is matched.

#### 2.2.3. Reconstructing Superimposable Substructures from High-Scoring Atom Pairs

To detect similar substructures, we apply a hierarchical clustering of atom pairs based on their mutual distances in 3D space [20]. In this procedure, two neighborhoods *i* and *j* (e.g., the yellow and green neighborhoods in Figure 2), each containing atom pairs $\left(a_{1}^{i},b_{1}^{i}\right)$, …, $\left(a_{k}^{i},b_{k}^{i}\right)$ and $\left(a_{1}^{j},b_{1}^{j}\right)$, …, $\left(a_{m}^{j},b_{m}^{j}\right)$, are merged if the Pearson correlation coefficient between their respective interatomic distance vectors within structures A and B (i.e., $\left(a_{1}^{i},a_{1}^{j}),(a_{1}^{i},a_{2}^{j}\right)$, …, $\left(a_{k}^{i},a_{m}^{j}\right)$ and $\left(b_{1}^{i},b_{1}^{j}),(b_{1}^{i},b_{2}^{j}\right)$, …, $\left(b_{k}^{i},b_{m}^{j}\right)$), is the highest among all possible merges and the coefficient exceeds a predefined threshold. A correlation threshold of 0.85 was used.

#### 2.2.4. Scoring of 3D Alignments

**Figure 3:**
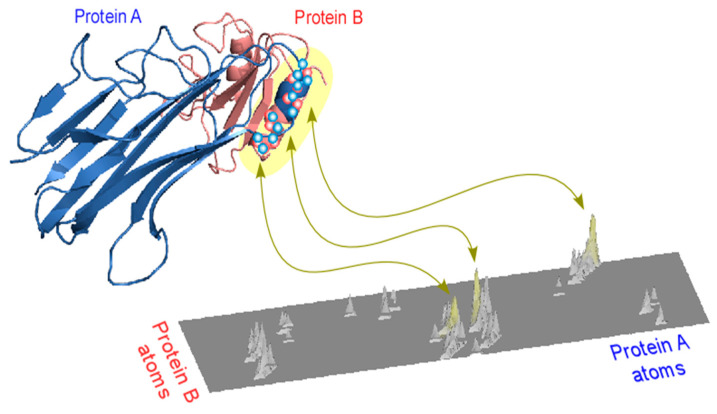
Schematic score plot of putative 3D superimposition. The heatmap illustrates alignment scores for atom pairs from two proteins, representing the likelihood that each pair participates in a valid 3D superimposition. Atoms from protein A are arranged arbitrarily along the *X*-axis, and atoms from protein B along the *Y*-axis. The score assigned to each atom pair reflects the cumulative quality of all of the linear alignments in which the pair appears. Light gray regions denote high-scoring atom pairs. Highlighted areas in yellow mark subsets of atom pairs that not only appear frequently in alignments but also preserve approximately equal all-against-all interatom distances within each protein, thereby defining a locally consistent 3D superimposable region.

Theoretically, a correlation coefficient close to 1 does not necessarily imply a small root mean square deviation (RMSD), which is a standard objective in geometric matching methods. For example, if one substructure is a scaled (proportionally expanded) version of the other, the correlation coefficient would be 1 despite a large RMSD. However, such cases are highly unlikely in real protein structures. Empirically, the correlation coefficient is well-correlated with the RMSD value of the superimposition: when the RMSD is zero, the correlation coefficient equals 1, and for small RMSD values, the correlation coefficient remains close to 1. Therefore, as a composite metric to assess both the significance and quality of geometric superimposition, we used the product of the number of matched atom pairs in the 3D alignment multiplied by the fifth power of the correlation coefficient (Ncorr5) (Figure 3). The use of the fifth power—rather than, for example, the third or seventh—was empirically determined based on experience, as it provided optimal separation between high-quality and spurious alignments in practice.

### 2.3. LigandPDB: Construction of a Reference Database of Known Ligand Neighborhoods

Protein structures from the PDB were split into the individual chains that comprise the entire protein structure or complex. For each ligand within a structure, a substructure was defined within each chain as a set of residues containing at least one atom within each of the residues located within 7 Å of any ligand atom. Only substructures of sufficient size (≥40 atoms) were retained as targets for 3D alignment with probe ligand cavities.

### 2.4. SurfXPDB: Construction of a Reference Database of Surface-Exposed Neighborhoods from the PDB

We used the FreeSASA 2.2.0 Python Module [21] to calculate the solvent-accessible surface area (SASA) for each target protein chain. Atoms with more than 2 Å^2^ of exposed area were classified as surface-exposed. For each such atom, all atoms belonging to residues that had at least one atom within 7 Å of it were selected as a substructure, which was then used for 3D alignment with the probe ligand cavities.

The computed SASA were added to the corresponding PDB files as a separate chain labeled ‘X’. Using a SurfX procedure, we then constructed the database of surface-proximal neighborhoods for all proteins in the PDB database.

### 2.5. Free-Energy Estimation and Ligand Binding Optimization with AutoDock Vina

The 3D superimposition of the probe ligand substructure in the query PDB file against a substructure of the target cavity in the target PDB file, according to the aforementioned 3D alignment, requires translocation/rotation of the target cavity in relation to the probe ligand substructure using the Kabsch algorithm [22]. After this superimposition, we performed a local optimization of the probe ligand position with respect to target cavity using the AutoDock Vina software [23]. As a result, the free-energy (FE) value is calculated for the optimized arrangement of probe ligand and the target protein cavity.

### 2.6. Benchmark Probe Dataset: Kahraman-36 Dataset

To evaluate SLAM’s performance, we used a widely recognized benchmarking dataset known as the Kahraman-36 dataset [2]. This dataset consists of 100 experimentally validated protein–ligand complexes, each bound to one of nine ligands: adenosine triphosphate (ATP), adenosine monophosphate (AMP), phosphate ion (PO_4_), alpha-D-glucopyranose (GLC), flavin adenine dinucleotide (FAD), ferroheme (HEM), flavin mononucleotide (FMN), ferroheme C (HEC), and beta-nicotinamide adenine dinucleotide (NAD).

This dataset was cross-referenced with the non-redundant set of crystalized protein structures available in the ProBiS database (nr-ProBiS) [24], which includes over 42,000 PDB entries. The intersection of the Kahraman-36 dataset and nr-ProBiS yielded 36 unique probe protein–ligand complexes. These 36 structures, referred to hereafter as ‘Kahraman36’, were used to generate a corresponding set of 36 probe ligand neighborhoods. This collection served as the benchmark for evaluating the performance of SLAM and ProBiS in detecting structural similarities between the probe proteins (Kahraman36) and the proteins in either the LigandPDB or SurfXPDB reference sets.

### 2.7. Threshold Selection for SLAM Scoring

#### 2.7.1. Determining the Threshold for Statistically Significant SLAM 3D Alignment Scores

SLAM scores are computed for each protein pair 3D-alignment, necessitating the definition of a significance threshold to distinguish meaningful structural matches from background noise. Since in SLAM the Ncorr5 scores of detected 3D alignments are maximized, we are modeling the score distribution using a double exponential Gumbel cumulative distribution function [25] for extreme values, defined as

$$F(x,\mu ,\beta )=e^{{-e}^{-\frac{x-\mu }{\beta }}},$$

where *F* is the cumulative probability of observing an extreme value *x*, and *μ*, *β* are parameters of the approximation.

Based on a match between empirical and Gumbel distributions, we established a threshold of 25 for the Ncorr5 alignment score. Namely, to construct an empirical background distribution for the Ncorr5 scores, we applied SLAM to each of the 36 protein–ligand complexes from Kahraman36, aligning them against the entire nr-ProBiS PDB database (~42,000 PDB protein–ligand structures). This comprehensive set of alignments provided a broad sample spanning both random and meaningful structural matches.

To validate the statistical threshold, we analyzed six subsets of randomly selected SLAM alignments, encompassing the full range of observed Ncorr5 scores. Five of these subsets contained 100,000 alignments each, and one contained 35,000 alignments. For each subset, we generated double-logarithmic plots for cumulative Ncorr5 score distributions.

**Figure 4:**
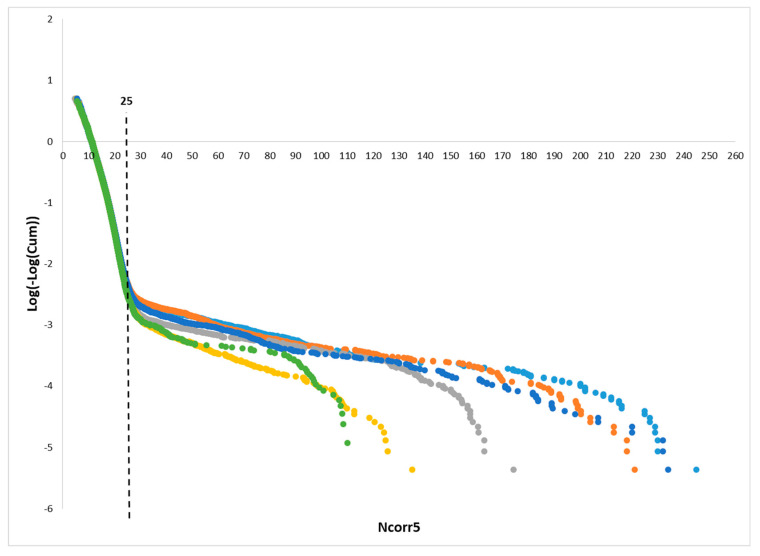
Determining the threshold for statistically significant SLAM 3D alignment scores. Six subsets of randomly selected SLAM alignments covering the full range of observed Ncorr5 scores were analyzed. Five subsets, each containing 100,000 alignments, are represented in yellow, gray, blue, orange, and light blue. A sixth subset, containing 35,000 alignments, is shown in green. For each subset, a double-logarithmic transformation of the cumulative distribution of Ncorr5 scores ([log(−log(Ncorr5))]) was plotted. Under the null hypothesis, these transformed distributions are expected to follow a Gumbel distribution, characteristic of extreme value statistics. A clear deviation among the empirical distributions and divergence from the expected Gumbel trend is observed at higher Ncorr5 values, indicating statistically significant alignments. Based on this analysis, a threshold of Ncorr5 ≥25 was determined to define significant SLAM 3D alignments.

The six double-log cumulative plots were nearly identical for Ncorr5 values below 25 (Figure 4), indicating that such scores conform well to the expected Gumbel distribution for random 3D alignments. However, for Ncorr5 scores above 25, the empirical distributions deviated from the Gumbel model, suggesting that such high scores represent statistically significant non-random alignments.

Based on this, we concluded that 25 is a significance threshold for SLAM alignments, but for comparative performance evaluations against ProBiS, we used a more stringent threshold of 30 to ensure that only the most reliable SLAM alignments were considered.

#### 2.7.2. Determining the Free-Energy Score Threshold for True-Positive Probe Ligand Docking

As aforementioned, the superimposition of the two protein substructures, based on 3D atom alignment carried out by either SLAM or ProBiS, is performed using the Kabsch algorithm [22] for translocation/rotation. This alignment establishes the positioning of a probe ligand neighborhood in protein A (query) in relation to the candidate binding site neighborhood in protein B (target). Consequently, this also establishes the position and orientation of the probe ligand with respect to the target protein binding site.

However, the resulting probe ligand’s position may not represent the optimal free-energy conformation within the target cavity. To refine this, we apply AutoDoc Vina [23] for local optimization of the probe ligand within the target cavity. Typically, this optimization results both in positional shift and an improvement in the binding free-energy estimate. A substantial shift in ligand position suggests that the original alignment-based transfer was suboptimal.

To quantitatively assess docking accuracy, we define a ‘free-energy score’ (FE-score) for the optimized ligand position:

FE-score = FE/(Shift + 0.1),

where FE is the binding free energy predicted by AutoDock Vina after optimization, Shift is the displacement between the original and optimized ligand positions (in Å), and 0.1 is a small regularization constant to prevent division by zero.

To establish a significance threshold for the FE-score metric, distinguishing true docking events from artifacts, we used SLAM to predict proteins that bind PFOA ligands. Amongst these SLAM-based predictions, some proteins are well-known PFOA binding proteins (e.g., albumin, transthyretin, PPARs, estrogen receptor α, and others; there are 641 PDB records of these proteins in different organisms). The FE-scores of these well-known PFOA binding proteins were used for establishing the significance threshold.

**Figure 5:**
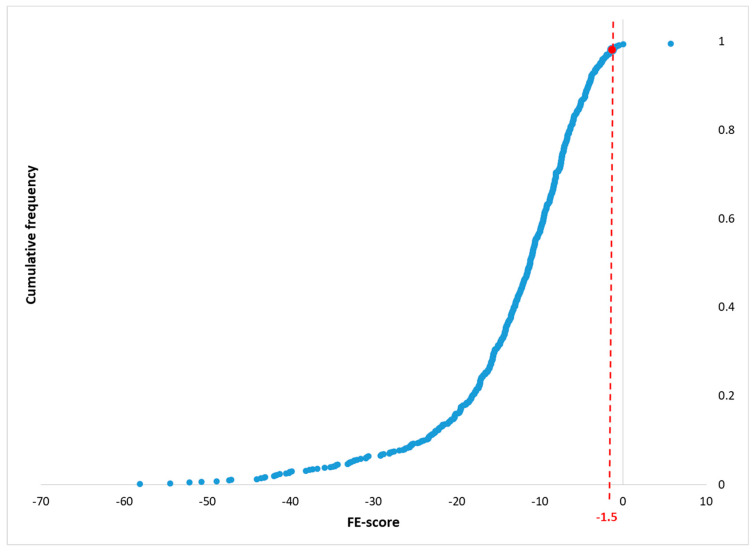
Establishing the free-energy score (FE-score) threshold for identifying true-positive probe ligand docking events. To determine a statistically supported threshold for the FE-score metric and distinguish true docking events from artifacts, we analyzed SLAM-based docking results for experimentally validated PFOA-binding proteins. The cumulative distribution of FE-scores across these known targets shows that 98% of the proteins exhibit FE-scores below −1.5. This empirical observation supports the use of −1.5 as a threshold for classifying docking results as true positives.

The cumulative distribution of these FE-scores (Figure 5) reveals that 98% of the experimentally known PFOA-binding proteins have an FE-score below −1.5, supporting the use of this threshold as a discriminator of true-positive docking events. Accordingly, the FE-score threshold for true-positive (TP) ligand docking was set to −1.5, so a protein with an FE-score < −1.5 is considered a potential good-quality docking site for a specific ligand.

### 2.8. Code Availability

All SLAM code is available from GitHub (Version 1): https://github.com/TauberBioinformatics/SLAM.

## 3. Results

### 3.1. Comparison of SLAM and ProBiS Algorithms Based on Ligand-Containing Cavities in Target Proteins

Performance of SLAM was compared to the popular ProBiS 2.0 ligand-docking software [10]. Similarly to SLAM, the ProBiS algorithm identifies putative probe ligand binding cavities in target proteins by comparing the structural features of the probe protein’s binding cavity with those of proteins in the target PDB database. This approach allows for detection of potential ligand-binding sites, even in cases where direct protein similarity is absent.

Since ProBIS does not differentiate between docking into cavities and solvent-exposed surfaces, whereas SLAM applies distinct approaches for each, we conducted separate comparisons of ProBIS and SLAM under each condition. Therefore, at first, we restricted ProBIS analysis to ligand-containing cavities in target proteins.

To compare SLAM and ProBiS, we utilized 36 protein–ligand crystallized structures from the Kahraman36 (Section 2.6), a widely accepted standard database for testing structural docking algorithms [2]. The 3D neighborhoods of these 36 probe ligand-binding sites were aligned against all target ligand-binding neighborhoods from the nr-ProBiS database [24], which includes 42,000 non-redundant proteins, including the Kahraman36 structures.

For SLAM, a 3D alignment was considered a good binding target if Ncorr5 >30 (see Section 2.7.1). For ProBiS, we selected all binding partners with a z-score ≥2 standard deviations (SDs). True-positive (TP) binding findings for both ProBiS and SLAM were defined as cases where FE-score < −1.5 after AutoDoc Vina local optimization (see Section 2.7.2).

**Figure 6:**
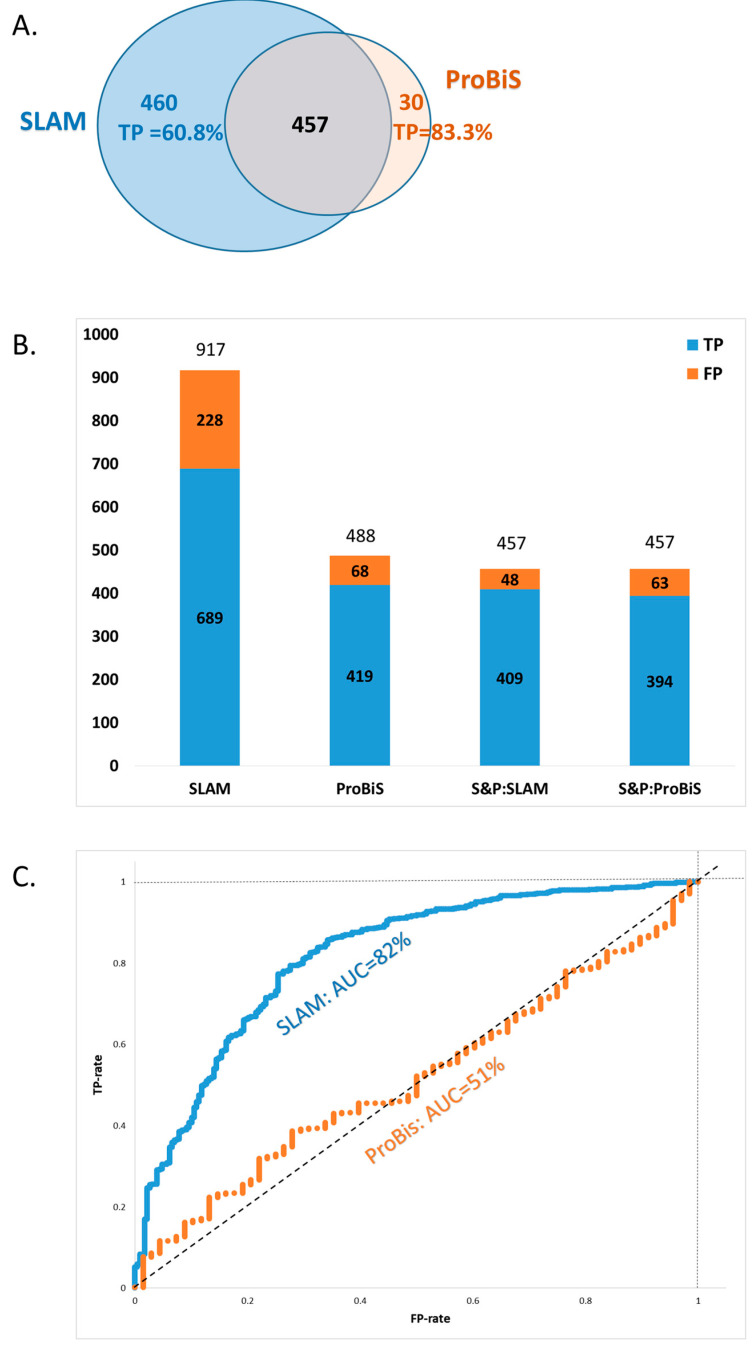
Comparative performance of SLAM and ProBiS in identifying ligand-binding protein partners using the Kahraman36 dataset and nr-ProBiS database. (**A**) Venn diagram showing the number of proteins identified by the SLAM and ProBiS algorithms and their overlap. Probe structures from the Kahraman36 dataset were aligned against the full nr-ProBiS database (~42,000 protein–ligand complexes). SLAM identified 460 unique protein pairs with Ncorr5 ≥ 30 that were not detected by ProBiS. From these, 280 (60.8%) were identified as TPs. Conversely, ProBiS identified 30 unique pairs with z-score ≥ 2 not found by SLAM. From these, 25 (83.3%) were identified as TPs. Both algorithms identified 457 overlapping protein pairs with significant 3D alignments based on their respective scoring thresholds. The threshold Ncorr5 ≥ 30 for SLAM was empirically determined to reliably capture biologically relevant neighborhood similarities. For ProBiS, z-score ≥ 2 is a standard cutoff indicating significant structural alignment, as supported by previous benchmarking studies and ProBiS documentation. (**B**) Stacked bar chart comparing the proportions of true positives (TPs) and false positives (FPs) identified by each algorithm. SLAM identified 917 potential binding proteins, of which 689 (75.1%) were classified as TPs (based on FE-score < −1.5), and 228 as FPs. ProBiS identified 488 proteins, including 419 TPs (85.9%) and 69 FPs. For the 457 proteins identified by both algorithms, results are shown separately based on the source of the ligand’s initial positioning used in FE-score calculations. Using SLAM-derived positions (‘S&P:SLAM’), 409 proteins (89.5%) were TPs and 48 FPs; using ProBiS-derived positions (‘S&P:ProBiS’), 394 were TPs (86.2%) and 63 FPs. (**C**) Receiver Operating Characteristic (ROC) curves illustrating the performance of the SLAM and ProBiS scoring metrics in distinguishing TPs from FPs. SLAM’s Ncorr5 score exhibits strong predictive value with an area under the curve (AUC) of 0.82. In contrast, ProBiS’s z-score shows limited discriminatory power, with an AUC of 0.51, suggesting near-random association with binding relevance.

In total, SLAM identified 917 target proteins as potential probe ligand binding partners (Ncorr5 > 30); of these, 460 proteins were not detected by ProBiS 2.0 (Figure 6A, Supplementary Table S1). These 3D alignments indicate structural similarity between ligand-binding cavities in the Kahraman36 dataset and those in target proteins from the nr-ProBiS database. From all 917 SLAM-identified proteins, 689 (75%) were TPs (FE-score < −1.5), while others did not have sufficiently good FE-scores indicating that these found binding partners were most likely not true bindings (Figure 6A). From the 460 proteins that were detected only by SLAM and not by ProBiS, 280 (60.8%) were classified as TPs. The remaining predicted binding partners had insufficient FE-scores, suggesting either a substantial shift in ligand position was required during docking or the optimized binding free energy was unfavorable (Figure 6A).

In total, ProBiS identified 488 proteins, 30 of which were not detected by SLAM (Supplementary Table S2). For these proteins, 25 (83.3%) were identified as TPs (FE-score < −1.5) (Figure 6A).

Among the identified proteins that bind Kahraman36 ligands within their ligand cavities, 457 were detected by both ProBiS and SLAM (Figure 6B, Supplementary Table S3). For these proteins, identified as potential binding partners by both algorithms, the likelihood of them being TP was high—exceeding 86%. It is important to note that FE-score calculations were performed separately for SLAM and ProBiS, as the initial ligand placements provided by the two algorithms were not always identical. As a result, the subsequent AutoDock Vina optimizations often differed. Within this shared set of proteins, the TP rate was 89.5% for SLAM and 86% for ProBiS (Figure 6B).

In order to estimate the predictive performance of SLAM against ProBiS, we performed a comparison of Receiver Operating Characteristic (ROC) curves for the two algorithms, which was conducted as follows: the best 3D alignments were ranked based on their alignment scores (z-score for ProBis and Ncorr5 for SLAM). The TP and false-positive (FP) rates were calculated separately for each algorithm (Figure 6C).

The ROC curves of the two algorithms demonstrate that TPs are strongly associated with SLAM’s Ncorr5 score, with an Area Under Curve (AUC) of 0.82. In contrast, the association of TPs with ProBiS’ z-score is weak, with an AUC of 0.51, indicating an almost random association (Figure 6C).

### 3.2. Comparison of the SLAM and ProBiS Algorithms for Docking Kahraman36 Probe Ligands into Solvent-Exposed Surface Patches of Target Proteins

Known ligand-binding cavities of the protein are not the only possible locations where a protein can bind the ligand. The solvent-exposed surface of a protein refers to the parts of the protein that are accessible to surrounding molecules of the solution. It includes the outermost atoms of the protein that are not buried inside the core of the structure. Thus, many algorithms that search for a potential binding of ligands to a protein consider solvent-exposed surface of the protein as potential binding sites.

Several algorithms are available for extracting the solvent-exposed surface of a protein [26,27,28]. In this study, we used the FreeSASA Python module [21,28] to generate a comprehensive collection of solvent-exposed surface patches for the entire nr-ProBiS database [24], resulting in the SurfaceXDB (Section 2.4).

We then used the 36 crystallized protein–ligand structures from the Kahraman36 dataset (Section 2.6) as probe ligands to screen the SurfXPDB of the nr-ProBiS database for potential binding patches. Again, we performed the screening using both SLAM and ProBiS algorithms, for a direct comparison of their performance.

For each probe ligand protein versus target protein comparison, only one best binding location was considered. In the SLAM and ProBiS analyses, the best result was a location with the lowest FE-score.

**Figure 7:**
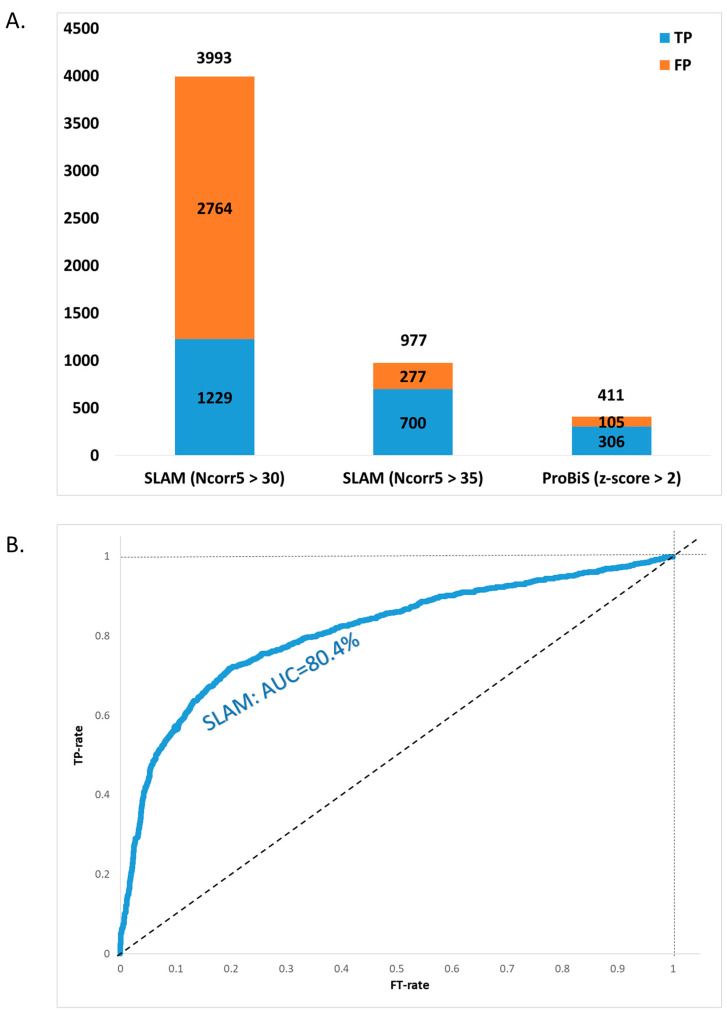
Comparison of SLAM and ProBiS algorithms for identifying ligand-binding sites on solvent-exposed surface patches. For each probe–target protein comparison, only the best predicted binding site (i.e., with the lowest FE-score) was considered. (**A**): Stacked bar chart showing the proportions of true positives (TPs) and false positives (FPs) identified by SLAM and ProBiS. SLAM identified 3993 potential binding partners with Ncorr5 > 30, of which 1229 (30.7%) had an FE-score < −1.5 and were classified as TPs (blue), while 2764 were classified as FPs (orange). When the Ncorr5 threshold was raised to 35, the number of TPs improved to 700 (71.6%), and the number of FPs dropped to 277, indicating improved specificity at higher score thresholds. ProBiS identified 411 binding partners with z-score > 2 (column: ‘ProBiS (z-score > 2)’), of which 306 (74.6%) were TPs and 105 were FPs, based on the same FE-score criterion. (**B**): Receiver Operating Characteristic (ROC) curve evaluating the ability of SLAM’s Ncorr5 score to distinguish TPs from FPs. The area under the ROC curve (AUC) is 0.804, indicating the strong predictive performance of Ncorr5 for assessing binding relevance.

SLAM identified a total of 3993 putative docking events with Ncorr5 > 30. Among these, only 30.7% were classified as TPs (FE-score < −1.5) (Figure 7A). However, increasing the Ncorr5 threshold to 35 resulted in 977 hits, of which 71.6% were TPs (Supplementary Table S4). This is due to a correlation between higher Ncorr5 scores and true-positive docking predictions based on FE-scores (Supplementary Figure S1), consistent with the trend observed in the SLAM ROC curve (Figure 7B).

Using the same Kahraman36 probe ligand proteins, ProBiS identified 411 hits with z-score ≥ 2, of which 74.6% were classified as TPs based on FE-score < −1.5 (Figure 7A, Supplementary Table S5).

Unlike the comparison of probe ligand cavities with target ligand-bound cavities (see Section 3.1), evaluating algorithm performance across the entire exposed surface is more complex, as SLAM and ProBiS assign scores to potential binding patches using fundamentally different methodologies. The results above indicate that SLAM identifies a greater number of true-positive cases (FE-score < −1.5). However, the SLAM-detected patches cannot be directly compared to the ProBiS-detected patches because only the top-scoring surface ligand-binding patches are taken into account for each protein and these may differ between the two methods.

### 3.3. Case Study: Identifying Ligand Inhibitors for CRISPR-Cas System Using the SLAM Algorithm

CRISPR-associated (Cas) proteins are key components of the CRISPR (Clustered Regularly Interspaced Short Palindromic Repeats) adaptive immune system in bacteria and archaea [29,30,31]. These proteins enable prokaryotic organisms to recognize and cleave foreign genetic material, such as bacteriophage DNA, thereby providing immunity against infections. Owing to their ability to introduce site-specific double-stranded breaks in DNA guided by programmable RNA sequences, Cas proteins have become powerful tools in biotechnology and medicine [29]. Their applications span genome editing, gene therapy, molecular diagnostics, and synthetic biology.

Ongoing research efforts aim to refine the mechanisms of Cas proteins, enhance their target specificity, and develop small-molecule inhibitors to improve control over genome-editing processes [32]. In parallel, there is increasing interest in identifying multi-Cas inhibitors—broad-spectrum compounds capable of modulating a range of CRISPR-Cas systems. Such inhibitors could act as universal off-switches, improving gene-editing safety by reducing off-target activity. Additionally, they hold promise for antimicrobial applications, facilitating streamlined workflows involving multi-Cas effectors, and deepening our understanding of CRISPR system biology [33,34].

We used a curated list of CRISPR–Cas complexes from Jia et al. [35] to retrieve corresponding PDB structures for further analysis. The resulting dataset included 10 Cas protein complexes bound to various ligands such as small molecules, RNA, and DNA (Supplementary Table S6). These served as the target set. As queries, we used all PDB entries containing small-molecule ligands collected in the LigandPDB database (see Section 2.3). This allowed us to systematically assess whether any known PDB ligands could potentially bind to Cas proteins at functionally relevant sites and thereby act as candidate Cas inhibitors.

From the 10 PDB entries containing Cas proteins, we compiled a database of Cas ligand neighborhoods, cavities in Cas proteins that interact with other CRISPR-Cas system components, RNA/DNA molecules, or known inhibitors. These include three families of proteins: Class I Cas proteins, which operate as multi-protein effector complexes, in which we identified interaction regions with guide RNA (gRNA), DNA, and other Cas subunits; Class II Cas proteins, which function as single-protein effectors, where we mapped contact regions with CRISPR RNA (crRNA) and DNA; and anti-CRISPR (Acr) proteins, which naturally inhibit CRISPR-Cas activity, and for which we extracted their contact sites with Cas proteins. Acr proteins are of specific interest, as their interaction regions may serve as potential binding sites for small molecules that could either block Acr-mediated interference or directly inhibit CRISPR-Cas function by mimicking Acr protein mechanisms. This collection of interaction sites was used as an input of probe proteins for the SLAM screening approach.

Using SLAM, we calculated alignment scores for all pairs of probe and query protein cavities and generated a list of potential Cas-binding ligands. Next, the high-quality alignments (Ncorr5 > 30) were further evaluated by assessing the FE of the binding between the Cas protein and the probe ligand, followed by FE-score calculation (see Section 2.7). Thus, we generated a list of the high-quality alignments with Ncorr5 > 30 and FE-score < −1.5 (Supplementary Table S7).

**Figure 8:**
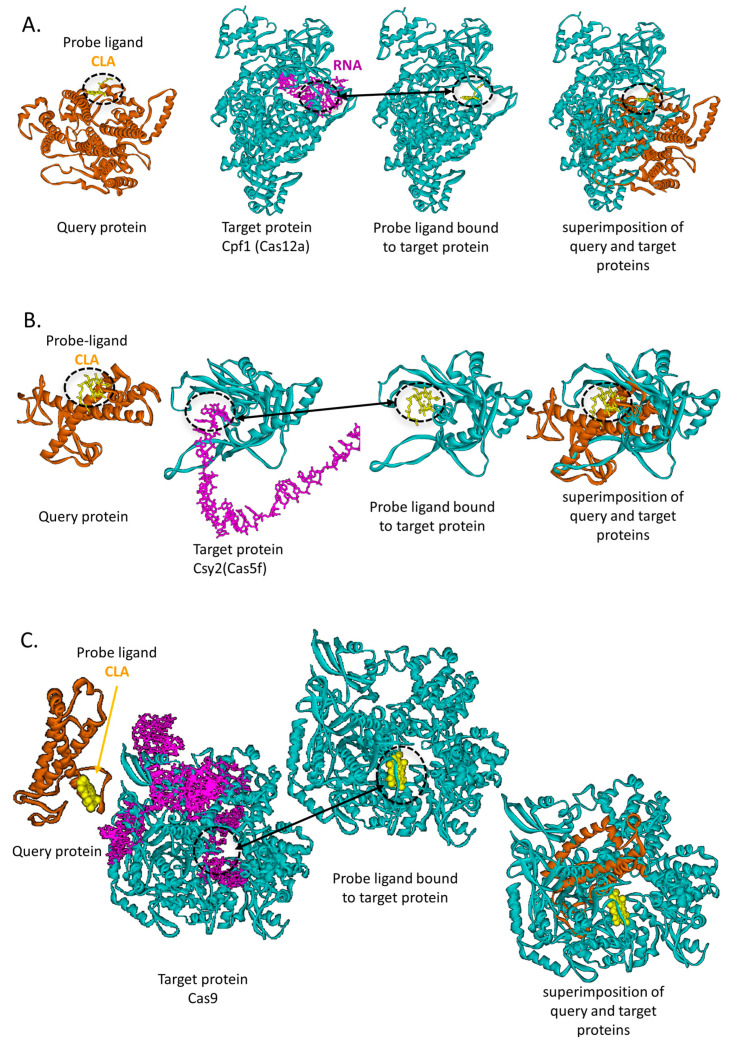
The CLA ligand is a potential multi-Cas-binding ligand. (**A**) Cpf1 (Cas12a): SLAM identified that CLA potentially binds to Cpf1 at the Cas–RNA binding interface with an Ncorr5 score of 36.95 and an FE-score of −4.96, suggesting potential interference with Cpf1 activity. The query protein (PDB ID: 8jw0, chain A) is shown in brown, and the probe ligand (CLA) in yellow. The target protein (PDB ID: 6nm9, chain D), encoding Cpf1 (Cas12a), is shown in light blue, and the associated RNA molecule (6nm9, chain E) in purple. Panel 1: Query protein bound to native ligand; Panel 2: Target protein with functional RNA interface; Panel 3: Predicted CLA-binding pose from SLAM refined with AutoDock Vina; Panel 4: Structural superimposition of query and target proteins with CLA. (**B**) Csy2 (Cas5f): SLAM identified that CLA potentially binds to Csy2 at the Cas–RNA binding interface with an Ncorr5 score of 37.97 and an FE-score of −4.25, suggesting potential interference with Csy2 activity. The query protein (PDB ID: 8jw0, chain J) is shown in brown, CLA in yellow, the target protein (PDB ID: 6whi, chain B) in light blue, and the RNA molecule (6whi, chain M) in purple. Panel layout as in (**A**). (**C**) Cas9: SLAM identified that CLA potentially binds to Cas9 at the Cas–RNA binding interface with an Ncorr5 score of 37.6 and an FE-score of −6.71, suggesting potential interference with Cas9 activity. The query protein (PDB ID: 8wb4, chain 4) is shown in brown, CLA in yellow, the target protein (PDB ID: 5f9r, chain B) in light blue, and the RNA molecule (5f9r, chain A) in purple. Panel layout as in (**A**).

Interestingly, our analysis identified several small molecules that may competitively interfere with several Cas complexes at their interaction sites with RNA or with Acr proteins (Supplementary Table S8). These candidate ligands exhibited favorable binding predictions, with FE-scores below −3, suggesting potentially strong interactions. Notably, a subset of these ligands was predicted to interfere at the Cas–RNA interface of several Cas proteins. For example, Chlorophyll A (PDB ligand ID: CLA) molecule was predicted to bind CPF1 (also known as Cas12a), CSY2 (also known as Cas5f), and Cas9 proteins at their RNA-binding cavities (Figure 8). Ethylene glycoledo (PDB ligand ID: EDO) was predicted to bind CPF1 and Cas9 at the RNA-binding interface; similarly, sulfate ion (PDB ligand ID: SO4) was also predicted to bind both Cas9 and CPF1 proteins at their interaction sites with RNA molecules. Interestingly, three small molecules (chlorophyll A: CLA, heme: HEM, and cholesterol hemisuccinate: YO1) were predicted to bind both Cas9 and CSY3 (also known as Cas7f) at their interaction sites with Acr proteins.

As the abovementioned examples illustrate, SLAM predicts which ligands bind specific proteins. In the case of Cas proteins, it is possible that some of these predicted ligands interfere with RNA or Acr binding to several Cas proteins, suggesting their role as multi-Cas inhibitors or modulators.

### 3.4. Case Study: Identifying PFOA and PFOS Binding Partners Using SLAM

Per- and polyfluoroalkyl substances (PFASs), including perfluorooctanoic acid (PFOA) and perfluorooctane sulfonate (PFOS), are widely recognized for their toxicity and persistence in the environment, and are known as ‘forever chemicals’ [36,37]. PFOA and PFOS are the two most-abundant PFASs found in the environment [38] and are linked to a range of adverse health effects, including liver damage [39], kidney disease [40,41], thyroid disease [42,43], immune system dysfunction [44,45], and an increased risk of certain cancers, particularly kidney and testicular cancer [46,47]. While we know of some proteins that interact with PFOS and PFOA, including albumin, peroxisome proliferator-activated receptors (PPARs), and fatty acid-binding proteins (FABPs) [44,48,49,50], the toxicity profile of PFASs is hard to explain based on these interactions alone, and it is assumed that they have multiple other interactions with additional proteins.

Utilizing the known crystallized structures of protein complexes with PFOA and PFOS, we screened the entire PDB to determine additional potential PFOA- and PFAS-binding proteins.

#### 3.4.1. Potential PFOA Binding Proteins Identified by SLAM-Based Approach

To identify potential PFOA-binding proteins, we selected experimentally resolved PDB structures containing PFAO as probe ligand complexes. These included human transthyretin bound to PFOA (PDB ID: 5JID), sea bream transthyretin complexes (PDB ID: 6GOO and 6GON), fatty acid-binding protein 3 (FAB3) in complex with PFOA (PDB ID: 7FEK), and the PPARγ receptor complexed with PFOA (PDB ID: 8U57). Ligand neighborhoods were extracted from each of these structures and used as probes. These were then screened against the LigandPDB database of protein ligand neighborhoods (Section 2.3) to identify proteins with locally similar binding cavities.

**Figure 9:**
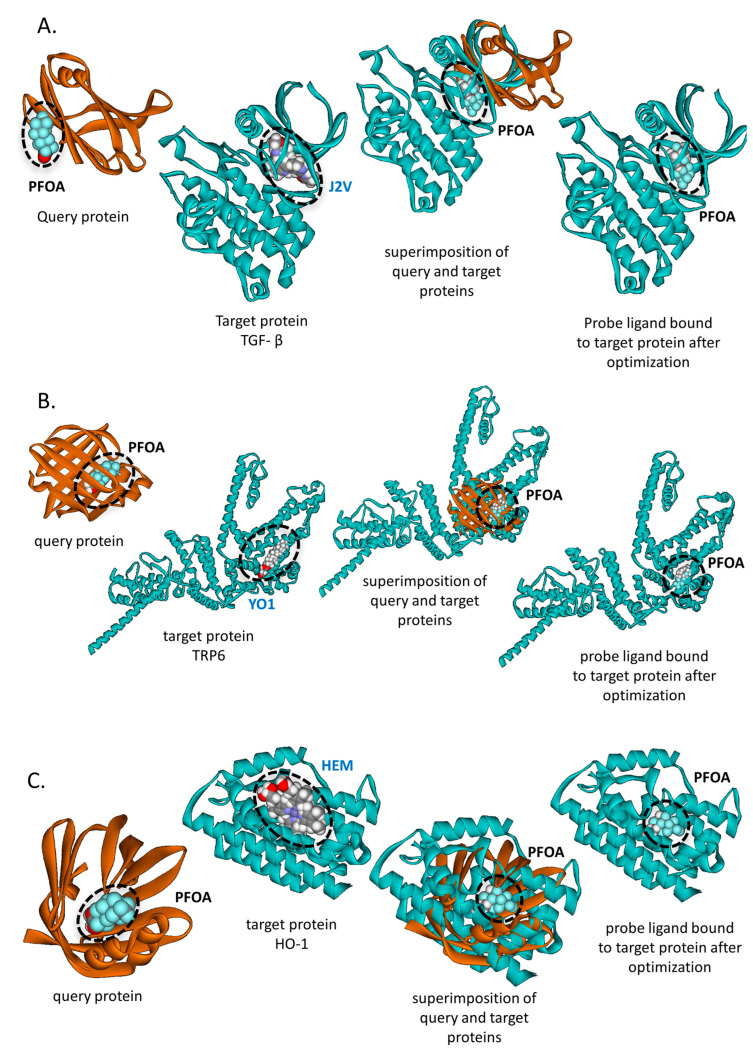
Potential PFOA-binding proteins identified by SLAM-based approach. (**A**) TGF-β: SLAM identified that PFOA potentially binds to TGF-β at the binding cavity of J2V, a known TGF-β inhibitor, with an Ncorr5 score of 33.26 and an FE-score of −5.61. The query protein (PDB ID: 6gon, chain A) is shown in brown, and the probe ligand (PFOA) in yellow. The target protein (PDB ID: 5qin, chain A), encoding TGF-β, is shown in light blue. Panel 1: Query protein bound to its native ligand (PFOA); Panel 2: Target protein with native ligand J2V; Panel 3: Structural superimposition of query and target proteins with PFOA at the respective binding site; Panel 4: Predicted PFOA-binding pose in the target protein (SLAM + AutoDock Vina). (**B**) TRP6: SLAM identified that PFOA potentially binds to TRP6 at the cholesterol (YO1) binding site, with an Ncorr5 score of 33.04 and an FE-score of −6.68, suggesting possible interference with TRP6 activity. The query protein (PDB ID: 7fek, chain A) is shown in brown, PFOA in yellow, and the target protein (PDB ID: 7dxg, chain A), encoding TRP6, in light blue. Panel layout as in (**A**), with Panel 2 showing the native ligand YO1. (**C**) HO-1 (Heme Oxygenase 1): SLAM identified that PFOA potentially binds to HO-1 at the heme (HEM) ligand cavity, with an Ncorr5 score of 33.53 and an FE-score of −6.76. The query protein (PDB ID: 7fek, chain A) is shown in brown, PFOA in yellow, and the target protein (PDB ID: 4g98, chain A), encoding HO-1, in light blue. Panel layout as in (**A**), with Panel 2 showing the native ligand HEM.

Using a SLAM-based approach, we identified 1930 proteins as potential PFOA-binding partners based on a FE-score threshold of <−1.5 (Supplementary Table S9). While some of these proteins are already known to interact with PFOA, many have not been previously reported. Notably, our results suggest that PFOA may bind to transforming growth factor beta (TGF-β PDB ID: 5QIN, chain A). Although no direct interaction between PFOA and TGF- β has been documented, prior studies have reported indirect effects of PFOA on TGF-β signaling [51,52]. SLAM predicts that PFOA occupies the ligand-binding cavity of TGF- β, which in the original PDB structure is bound by N-{4-[3-(6-methoxypyridin-3-yl)-1H-pyrrolo[3,2-b]pyridin-2-yl]pyridin-2-yl}acetamide (J2V), a known TGF- β inhibitor (Figure 9A). This suggests that PFOA may directly interfere with TGF-β activity by competitively binding to its functional site.

Another protein identified as a potential PFOA-binding target using SLAM is Transient Receptor Potential Channel 6 (TRPC6). Specifically, SLAM analysis revealed that the cholesterol hemisuccinate (YO1)-binding site in TRPC6 shares significant local 3D similarity with cavities from other proteins known to bind PFOA, suggesting that TRPC6 may also accommodate PFOA within this region. TRPC6 is a key regulator of intracellular calcium homeostasis and has been implicated in a range of pathological conditions, including increased vascular endothelial permeability, cardiac pathology, renal dysfunction and various cancers [53,54,55,56]. Its activity is modulated by cholesterol and other endogenous lipids [57], raising the possibility that PFOA, due to its structural resemblance to lipophilic molecules, may similarly effect TRPC6 channel activity (Figure 9B).

Lastly, the enzyme heme oxygenase-1 (HO-1), which catalyzes the degradation of heme, was also identified by the SLAM-based approach as a potential PFAO-binding protein. The analysis suggests that PFOA may occupy the heme-binding cavity of HO-1, potentially interfering with its enzymatic activity (Figure 9C). HO-1 is strongly induced in most cell types under oxidative stress as a protective mechanism to prevent reactive oxygen species (ROS)-induced damage. Inhibition of HO-1 activity could therefore result in ROS accumulation and increased oxidative stress. While PFOA has been reported to modulate HO-1 expression, the underlying mechanism is still not completely established and may involve both direct and indirect pathways [58,59].

#### 3.4.2. Potential PFOS-Binding Proteins Identified by SLAM-Based Approach

**Figure 10:**
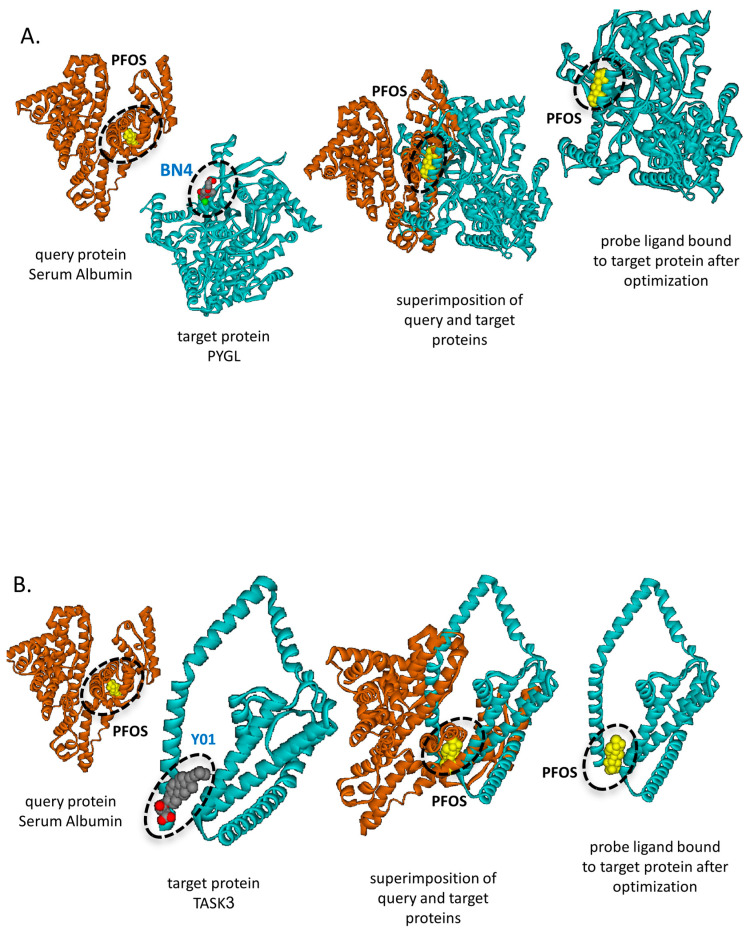
Potential PFOA-binding proteins identified by SLAM-based approach. (**A**) PYGL: SLAM identified that PFOS potentially binds to PYGL at the binding cavity of BN4, with an Ncorr5 score of 36.628 and an FE-score of −4.017. The query protein (PDB ID: 4e99, chain A) is shown in brown, and the probe ligand (PFOS) in yellow. The target protein (PDB ID: 1wv0, chain A), encoding PYGL, is shown in light blue. Panel 1: Query protein bound to its native ligand (PFOS); Panel 2: Target protein with native ligand YO1; Panel 3: Structural superimposition of query and target proteins with PFOS at the respective binding site; Panel 4: Predicted PFOS-binding pose in the target protein (SLAM + AutoDock Vina). (**B**) TRP6: SLAM identified that PFOS potentially binds to TASK3 at the cholesterol (YO1) binding site, with an Ncorr5 score of 33.306 and an FE-score of −4.02, suggesting possible interference with TASK3 activity. The query protein (PDB ID: 4e99, chain A) is shown in brown, PFOA in yellow, and the target protein (PDB ID: 8k1q, chain A), encoding TASK3, in light blue. Panel layout as in (**A**), with Panel 2 showing the native ligand YO1.

Only one crystallized complex containing PFOS is currently available in the PDB: the human serum albumin structure (PDB ID: 4E99). This structure was used to generate a single PFOS probe ligand substructure. Using SLAM, we identified 147 potential PFOS-binding proteins with FE-scores below −1.5. (Supplementary Table S10). Notable examples include liver glycogen phosphorylase (PYGL), the key enzyme responsible for glycogen breakdown in the liver [60,61] (Figure 10A). Previously, it has been shown that PFOS decreases liver glycogen content and increases hepatic lipid accumulation [62]. Another predicted PFOS-binding protein is TASK3 (encoded by KCNK9), a potassium channel predominantly active in neurons regulating resting membrane potential and excitability [63,64,65] (Figure 10B).

## 4. Discussion

The SLAM algorithm introduces a novel framework for detecting local 3D similarities between protein structures. It represents each protein structure as a linear collection of overlapping atomic neighborhoods, with each neighborhood encoded as a short sequence of heavy atoms ordered by spatial proximity to a central heavy (non-hydrogen) atom and each atom is annotated with physicochemical features. These sequences are aligned using the Needleman–Wunsch algorithm to identify recurring atom-level matches. Frequently occurring high-scoring alignments reveal candidate atom pairs, which are then clustered into larger substructures by optimizing the Pearson correlation of interatomic distances. This yields structurally consistent local alignments suitable for ligand transfer and docking.

For assessing SLAM efficiency, we compared SLAM to one of the most widely used tools for finding structural similarities between ligand-binding regions of proteins, ProBiS [10,66,67]. ProBiS compares the ligand-binding cavity of a query protein to either known ligand-binding cavities of a target protein or searches for structurally similar patches on the solvent-accessible surface of the target protein. For each target protein, ProBiS returns an expectation value (e-value) based on the Karlin–Altschul equation [68]. These values reflect the local similarity between the query ligand-binding cavity and each patch in the target protein, thereby indicating the likelihood of the probe ligand binding to a target protein. To compare SLAM to ProBiS, we used the ‘Kahraman36’ benchmarking dataset of probe ligand cavities [2]. We screened PDB proteins for structural similarity to the ligand cavities in Kahraman36 and found that SLAM identified a greater number of potential binding partners for these ligands with a higher TP rate (Figure 6), as supported by the AutoDock Vina free-energy calculations. It should be noted that this evaluation is based on docking-derived estimates and does not include experimental validation; therefore, the reported true-positive results represent computational predictions rather than confirmed biological interactions. SLAM also demonstrated strong performance in identifying potential binding sites when comparing probe ligand cavities to the solvent-exposed surfaces of target proteins. While direct comparison with ProBiS was complicated by fundamental differences in how each algorithm scores potential surface cavities, our analysis shows that SLAM consistently identified a larger number of potential binding partners. Importantly, SLAM’s scoring metric was positively correlated with the TP rate of binding site predictions, as evidenced by the ROC curve analysis (Figure 7), underscoring its predictive reliability in this more challenging search context.

To demonstrate SLAM applicability to different research analysis scenarios, we examined SLAM’s performance in two-way prediction. One is to predict ligands that bind to specific proteins, and the other is to predict the proteins that bind specific ligands.

SLAM’s applicability for identifying ligands that bind specific proteins was examined by searching for potential inhibitors of various CRISPR-Cas systems by targeting their key interaction sites, specifically Cas-single guide RNA (sgRNA) and phage-encoded Cas-anti-CRISPR (Acr) interfaces. First, we extracted these functional sites from known PDB entries of CRISPR-Cas complexes. Next, using SLAM, we screened all PDB ligand-binding protein cavities for similarities with CRISPR-Cas functional sites. Thus, SLAM analysis revealed several candidate ligands that can competitively bind to functional sites of Cas proteins with good FE-scores, indicating potentially strong and specific interactions. Such ligands have the potential to act as Cas modulators. Importantly, a subset of these ligands recurred across multiple Cas family members, raising the possibility of developing broad-spectrum inhibitors (Figure 8, Supplementary Table S8). Such compounds could serve as valuable molecular tools for regulating CRISPR-Cas activity, enhancing the safety of genome-editing applications, and enabling finer temporal or spatial control over Cas-mediated processes.

To further demonstrate SLAM’s applicability in finding proteins that bind specific ligands, we applied SLAM to identify candidate binding proteins of perfluorooctanoic acid (PFOA) and perfluorooctane sulfonate (PFOS). At first, we compiled a set of known PFOA- and PFOS-binding cavities from PDB structures and used them to query the entire PDB database for locally similar ligand-binding cavities in unrelated proteins. SLAM analysis revealed that many of the predicted ligand-binding proteins have been previously reported to be directly affected by PFAS exposure or are involved in molecular pathways influenced by these toxic compounds. For example, of the already known PFAS binding proteins, SLAM identified serum albumin [48], FABPs [50,69], several peroxisome proliferator-activated receptors (PPARs) [49,70], transthyretin, a thyroid hormone transport protein [71], and others.

Nonetheless, SLAM also identified novel proteins that may bind PFOS and/or PFOA and have not been previously reported. These proteins could potentially be affected by such interactions. For example, androgen receptor (AR) was shown to bind PFOA. This interaction was never shown previously, and only recently it was found that newer PFAS molecules, with structural resemblance to PFOA, such as 9-(nonafluorobutyl)-2,3,6,7-tetrahydro-1 H,5 H,11 H-pyrano[2,3-*f*]pyrido[3,2,1-ij]quinolin-11-one (NON), 2-(heptafluoropropyl)-3-phenylquinoxaline (HEP), and 2,2,3,3,4,4,5,5,5-nonafluoro-N-(4-nitrophenyl)pentanamide (NNN) directly bind the human AR and disrupt androgen-responsive gene expression [72]. To the best of our knowledge, this is the first indication that PFOA may also interact with AR. SLAM identified additional novel proteins that potentially bind PFOA, including TGF-β, which has previously been associated with PFOA only through indirect evidence (Figure 9A) [51,52], and TRPC6 (Figure 9B). TGF-β is a multifunctional cytokine belonging to the transforming growth factor superfamily and is the primary driver of fibrosis in most forms of chronic kidney disease [73]. Recent studies have shown that PFOA exposure significantly increases mRNA levels of TGF-β in mouse kidneys [51], possibly indicating a compensatory mechanism. TRPC6 is a key regulator of intracellular calcium homeostasis and has been implicated in a wide range of pathological conditions, including increased vascular endothelial permeability, cardiac dysfunction, renal disease, and multiple cancers [53,54,55,56].

SLAM also identified novel binding partners for PFOS including PYGL, a key enzyme responsible for glycogen breakdown in the liver [60,61] and TASK3, a potassium channel predominantly active in neurons regulating resting membrane potential and excitability [63,64,65] (Figure 10). In general, the identification of novel PFAS interactions by SLAM emphasizes potential SLAM applicability in environmental toxicology studies, since SLAM can be used for assessment of any pollutant’s toxicity and its potential targets.

The fact that SLAM identified known interactions strengthens the validity of this approach. Furthermore, while the abovementioned SLAM predictions of unknown interactions still require experimental validation, the overlap of SLAM-identified PFAS-binding proteins with biologically relevant known pathways and PFAS-associated pathological conditions enhances SLAM’s validity and suggests a potential mechanistic explanation for PFAS toxicity.

Furthermore, SLAM can be used in the pharmaceutical industry in two potential applications. One is to predict novel small molecules and drugs that will affect certain proteins of interest for their inhibition or activation. The other application is based on SLAM’s ability to predict unknown interactions of drugs with other proteins besides their targeted ones, thus predicting adverse effects that sometimes could be fatal. Accurate prediction of adverse effects in the early stages of drug development may guide safety evaluations and reduce the likelihood of later costly recalls, as well as mitigate the risk of serious, preventable illnesses in patients.

One of the advantages of SLAM is its flexibility and efficiency in finding correlated arrangements of atoms in two protein structures. This approach enables the screening of very large numbers of proteins for their similarities. Hence, one of the possible SLAM applications is the screening of the entire AlphaFold database consisting of more than two million structures for patches on the solvent-exposed surfaces fitted to the docking of ligands that are present in the protein–ligand complexes of the PDB data bank. Another advantage of SLAM is that its automated approach based on the alignment of short sequences requires less computational space and power, enabling the procedure to be performed using cheaper and simpler hardware.

Another significant extension of SLAM is that it can also be used for identifying novel protein–protein interactions based on previously known protein complexes that are registered in either the PDB or AlphaFold database. This could be achieved by separating the interacting surfaces into pairs of interacting patches and treating one of the patches as a ligand.

## 5. Conclusions

In conclusion, we propose SLAM as a novel robust approach for identifying potential protein–ligand interactions with many advantages and possible applications, both in environmental toxicology and in the pharmaceutical industry. We also showed that it can identify additional target proteins that were not found with the current approaches, with higher precision.

## Data Availability

All of the data generated or analyzed during this study are included in this published article and its Supplementary Information files.

## Supplementary Materials

The following supporting information can be downloaded at https://www.mdpi.com/article/10.3390/life16020285/s1, Figure S1: Correlation of SLAM NCorr5 and ProBis z-score with FE-score; Table S1: uniqueSLAM_ligand_460prot; Table S2: uniqueProBiS_30prot; Table S3: SLAM&ProBiS_ligand_457prot; Table S4: SLAM_surface_Ncorr5_35_977prot; Table S5: ProBis_AX; Table S6: CRISPR_Cas_target; Table S7: CRISPR_Cas_Results; Table S8: MultiCas_ligands; Table S9: PFOA_results; Table S10: PFOS_results.

## Abbreviations

The following abbreviations are used in this manuscript:

SLAM	Spacio-Linear Alignment of Macromolecules
PDB	Protein Data Bank
PFAS	Per- and Polyfluoroalkyl Substances
PFOA	Perfluorooctanoic Acid
PFOS	Perfluorooctane Sulfonate
ProBiS	Protein Binding Sites (Algorithm)
ATP	Adenosine Triphosphate
AMP	Adenosine Monophosphate
FAD	Flavin Adenine Dinucleotide
FMN	Flavin Mononucleotide
NAD	Nicotinamide Adenine Dinucleotide
HEM	Ferroheme
HEC	Ferroheme C
GLC	α-D-Glucopyranose
PO_4_	Phosphate Ion
SASA	Solvent-Accessible Surface Area
FE	Free Energy
FE-score	Free-Energy Score
RMSD	Root Mean Square Deviation
ROC	Receiver Operating Characteristic
AUC	Area Under the Curve
TP	True Positive
FP	False Positive
SD	Standard Deviation
nr-ProBiS	Non-Redundant ProBiS Database
LigandPDB	Database of Ligand-Binding Protein Neighborhoods
SurfXPDB	Database of Surface-Exposed Protein Neighborhoods
CRISPR	Clustered Regularly Interspaced Short Palindromic Repeats
Cas	CRISPR-Associated
Acr	Anti-CRISPR
gRNA	Guide RNA
crRNA	CRISPR RNA
PPAR	Peroxisome Proliferator-Activated Receptor
FABP	Fatty Acid-Binding Protein
TGF-β	Transforming Growth Factor Beta
TRPC6	Transient Receptor Potential Channel 6
HO-1	Heme Oxygenase 1
PYGL	Liver Glycogen Phosphorylase
TASK3	TWIK-Related Acid-Sensitive K^+^ Channel 3
AR	Androgen Receptor
