# Supplementary figures and images for: Spacio-Linear Screening for Ligand-Docking Cavities in Protein Structures: SLAM Algorithm
Source: Life (Basel). 2026 Feb 7;16(2):285. doi: 10.3390/life16020285 (PMC12942009; doi:10.3390/life16020285)

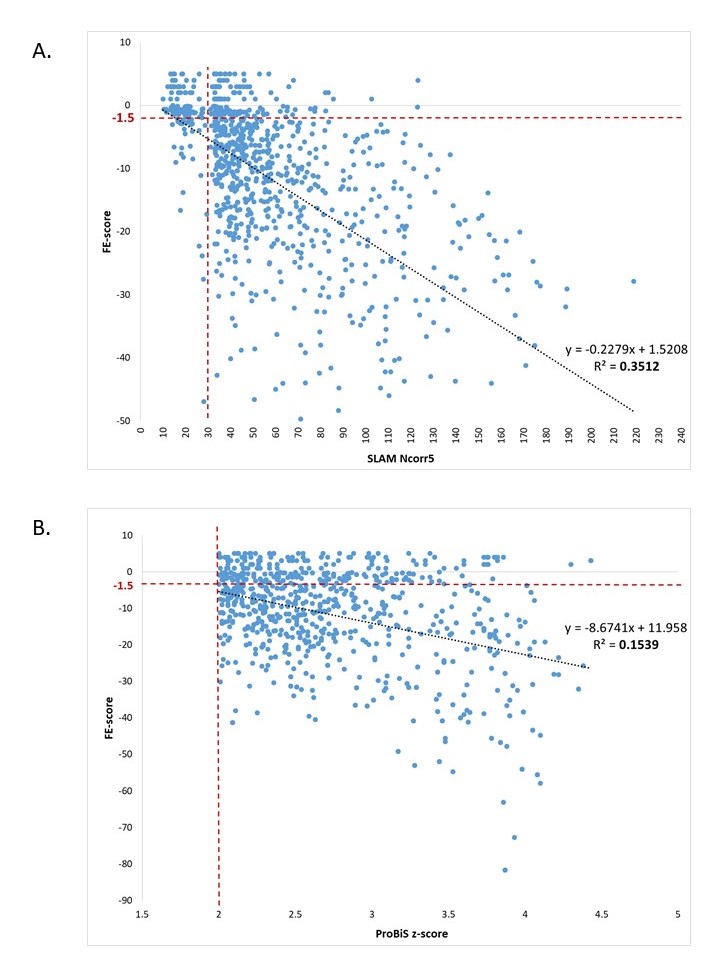

Supplement: Supplementary file 1 [file life-16-00285-s001.zip › Supp_Fig-S1.JPG]
